# Recovery Study of Gold Nanoparticle Markers from Lateral Flow Immunoassays

**DOI:** 10.3390/ma16175770

**Published:** 2023-08-23

**Authors:** Tilen Švarc, Peter Majerič, Darja Feizpour, Žiga Jelen, Matej Zadravec, Timi Gomboc, Rebeka Rudolf

**Affiliations:** 1 Faculty of Mechanical Engineering, University of Maribor, 2000 Maribor, Slovenia; peter.majeric@um.si (P.M.); z.jelen@um.si (Ž.J.); matej.zadravec@um.si (M.Z.); timi.gomboc@um.si (T.G.); rebeka.rudolf@um.si (R.R.); 2 Zlatarna Celje d.o.o., 3000 Celje, Slovenia; 3 Institute of Metals and Technology (IMT), 1000 Ljubljana, Slovenia; darja.feizpour@imt.si

**Keywords:** gold nanoparticles, recovery, LFIA, ultrasonic spray pyrolysis, characterisation

## Abstract

Lateral flow immunoassays (LFIAs) are a simple diagnostic device used to detect targeted analytes. Wasted and unused rapid antigen lateral flow immunoassays represent mass waste that needs to be broken down and recycled into new material components. The aim of this study was to recover gold nanoparticles that are used as markers in lateral flow immunoassays. For this purpose, a dissolution process with aqua regia was utilised, where gold nanoparticles were released from the lateral flow immunoassay conjugate pads. The obtained solution was then concentrated further with gold chloride salt (HAuCl_4_) so that it could be used for the synthesis of new gold nanoparticles in the process of ultrasonic spray pyrolysis (USP). Various characterisation methods including scanning electron microscopy, transmission electron microscopy, ultraviolet-visible spectroscopy and optical emission spectrometry with inductively coupled plasma were used during this study. The results of this study showed that the recovery of gold nanoparticles from lateral flow immunoassays is possible, and the newly synthesised gold nanoparticles represent the possibility for incorporation into new products.

## 1. Introduction

Nanoparticles are used in various industries and consumer products, such as electronics, cosmetics, medicine, food packaging and single-use diagnostic devices, due to their unique and useful properties. A problem arises when these nanoparticles are not managed properly at the end of their lifecycle and become waste [1]. Waste nanoparticles can pose significant challenges and risks to the environment and human health. This raises key concerns regarding their environmental impact, as they can enter the environment through various channels, including release from production processes, product use, disposal, by-products and mineral extraction. Their small size allows them to travel long distances, which represents a potential for polluting ecosystems [2,3]. Once the nanoparticles are in the environment, they can interact with organisms and ecosystems, potentially disrupting the local ecological balance [4,5]. These points of course raise concerns for human health. The small size of nanoparticles enables them to penetrate biological barriers and reach organs and tissues [6]. This raises concerns about potential health effects on humans, such as respiratory issues, cellular damage and inflammatory responses. The precise health risks associated with specific nanoparticles depend on their composition, shape, size and exposure levels. Proper disposal and treatment of waste nanoparticles are crucial to minimise their environmental and health impacts. However, many conventional waste management systems are not designed to handle nanoparticles effectively. Improper disposal routes, such as landfilling or incineration, may release nanoparticles into the environment or cause other harmful effects [7]. 

Advances in nanotechnology and the use of nanomaterials will also increase the amount of nanomaterial waste. As many of these are composed of high-value and scarce precious metals, some methods are being developed for their recovery. These methods may involve obtaining gold from gold nanoparticle suspensions with the use of HBr and α-cyclodextrin [8] or using commonly available reagents, such as NaCl and HCl in H_2_O_2_, in order to separate the gold from the laboratory waste, with a process involving reflux procedures [9]. The extraction of gold from larger solid wastes in the form of Au(0) or Au(III) is well established, both at the industrial and laboratory level. The most widespread processes are based on hydrometallurgical methods, which involve the use of nitric and hydrochloric acids in order to refine the gold, as well as toxic leaching solutions (including cyanides, halides, thiosulfates, thiourea, thiocyanate, etc.) [10]. More advanced gold extraction systems use ion exchange resins, which extract pure gold ions from solutions with gold [11], reducing losses of this precious metal further in the refining process.

The COVID-19 pandemic resulted in the accumulation of huge amounts of waste across Europe and the world. This was also related to the field of disposable medical devices, including rapid antigen LFIAs, which ensure fast, selective and reliable diagnoses of infected persons or virus carriers in various environments. LFIA work on the principle of immuno-chromatographic testing, based on the lateral traction of a liquid medium. Gold nanoparticles (AuNPs) are commonly used as markers in lateral flow immunoassays (LFIA) and are located on conjugate pads [12,13]. After use, LFIAs are sent for incineration. Based on our previous research [14], when burning one million LFIAs, 1 g of AuNPs and 5000 kg of plastic are thrown away, which represents a roughly estimated value of EUR 15,000. A literature review and theoretical calculations show that each kilogram of newly mined gold from low-Au ore leaves behind 16 tons of CO_2_, while a recycled kilogram of Au burdens the environment with only 53 equivalent kilograms of CO_2_ [15]. Therefore, after 2020, approximately 30% of the world’s gold supply was recycled from “old gold” rather than mined. On the other hand, a kilogram of plastic produced makes an average of 3 kg of CO_2_, which represents a high burden, while recycled plastic only releases 1 kg of CO_2_ equivalent [16,17,18]. Based on these facts, our idea was to study the reuse of AuNPs, as this would relieve the environment and affect the reduction of CO_2_ emissions indirectly. The current recycling technology for LFIAs used by Reworked from Great Britain does not allow the separation of individual components (AuNPs and white plastic), but for processing into secondary black plastic, the entire rapid test is used, which also contains AuNPs [19]. The Maribor University Clinical Centre (Slovenia) generated 1854 tons of waste in 2020, of which 276 tons were infectious waste and 2000 rapid antigen LFIAs per week. Waste management is an important part of the hospital’s activities, as it can be a source of hospital-acquired infections, which are considered one of the major public health problems in Slovenia [20].

Ultrasound spray pyrolysis (USP) is an upscale ready nanoparticle synthesis process that utilises a piezoelectric crystal for aerosol generation. The use of ultrasonic nebuliser results in a well-controlled particle size distribution [21] and energy efficiency [22]. Throughout the particle synthesis process, every precursor aerosol undergoes a series of physical and chemical transformations during the nanoparticle formation: solvent liquid evaporation, salt precipitation, pyrolysis and a reduction reaction [21,22]. Collection of the synthesised nanoparticles is carried out with electrostatic filters or liquid washing in collection bottles with stabilising agents [23]. These suspensions can be lyophilised to remove water and make dried particles, which can be used further in various applications where water is undesirable, including medical applications such as drug carriers or antibody conjugates [24,25], cosmetic creams [26] and polymer embedding [27].

The purpose of this study was to use AuNPs from unused LFIAs after their expiration date so that, in the next step, they would be suitable for the preparation of a solution that could serve as a starting material for the synthesis of new AuNPs in the USP process. The lyophilisation process was also used to obtain dried AuNPs, as they enable various applications [24,28,29,30,31,32]. Knowledge of such a process would contribute significantly to the circular economy in the field of nanomaterials, and as such, AuNPs could be reused in new products.

## 2. Materials and Methods

### 2.1. Recovery of AuNPS from Conjugate Pads

**Figure 1:**
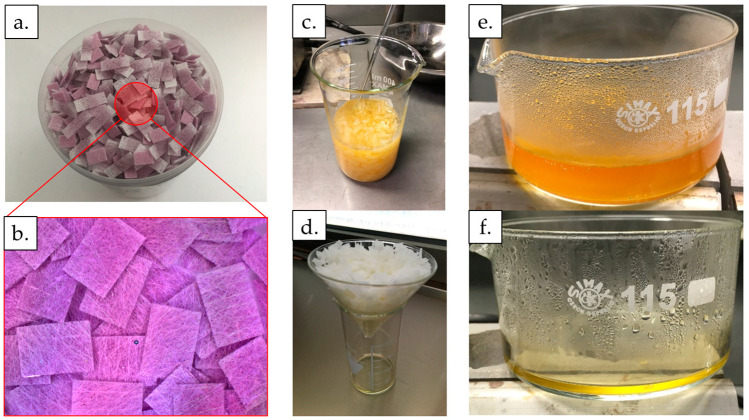
The process of dissolving AuNPs from the conjugate pads from LFIAs. (**a**) Conjugate pads with AuNPs. (**b**) Macro image showing conjugate pads with AuNPs. (**c**) Soaking the conjugate pads for 2 h in 200 mL of aqua regia. (**d**) Draining the soaked conjugate pads for 65 h. (**e**) Heating the aqua regia solution with leached gold ions at 70 °C for 75 min. (**f**) Aqua regia solution with leached gold ions after the evaporation of water and decomposition of excess aqua regia.

Conjugate pads with a total weight of 9.58 g were used for the recovery study of AuNPs from commercial LFIAs (Figure 1a,b). For dissolving AuNPs from the conjugate pads, 200 mL of aqua regia prepared from nitric acid (HNO_3_) and hydrochloric acid (HCl) were used in a volume ratio of 1:3. The conjugate pads were soaked for 2 h in the prepared aqua regia (Figure 1c). Extraction of AuNPs from conjugate pads followed the classical chemical reaction of Au dissolution in aqua regia, as described in Equation (1) [33].

Au + 4HCl + HNO_3_ → HAuCl_4_ + 2H_2_O + NO (1)

After soaking, the resulting solution was filtered for 65 h. In total, 150 mL of aqua regia was obtained, as some of it was soaked up by the conjugate pads and some water from the solution evaporated during the filtration process. This was followed by the process of heating the resulting solution at 70 °C for 1 h and 15 min down to a volume of 15 mL (Figure 1e,f) under a fume hood, due to the release of NOCl fumes from the decomposition of the excess aqua regia. Upon contact with oxygen in the air, the fumes decomposed into Cl_2_ and NO_2_ [34].

The used glassware was additionally rinsed with 5 mL of distilled water, which was added to the prepared Au solution to a final volume of 20 mL. The Au content was measured in both solutions, i.e., in the one that was formed immediately after soaking in the aqua regia and filtration and in the solution that was subjected to heating. Optical emission spectrometry with inductively coupled plasma (ICP-OES) was used for this purpose in order to prepare the starting solution (as a precursor) with an appropriate concentration of Au (at least 1 wt. %) in the USP process for the synthesis of new AuNPs.

#### Optical Emission Spectrometry with Inductively Coupled Plasma (ICP-OES) 

An HP, Agilent 7500 CE spectrometer, equipped with a collision cell (Santa Clara, CA, USA) was used to determine the Au content in the solutions. The following conditions were used for ICP-OES: the power was 1.5 kW, Nebuliser–Meinhard, the plasma gas flow was 15 L/min, the nebuliser gas flow was 0.85 L/min, the make-up gas flow was 0.28 L/min and the reaction gas flow was 4.0 mL/min. The instrument was calibrated with matrix-matched calibration solutions. The relative measurement uncertainty was estimated as ± 3%.

### 2.2. Synthesis of New AuNPs

#### 2.2.1. USP Synthesis

A solution (precursor) (V = 1 L) with a concentration of Au = 1 g/L was used for USP synthesis of new AuNPs. Gold chloride salt (HauCl_4_, Au content 50%, Glentham Life Sciences, Corsham, UK) was used for additional concentration of the solution obtained from the recovery process, as described in Section 2.1, so that the required Au concentration value was reached. 

**Figure 2:**
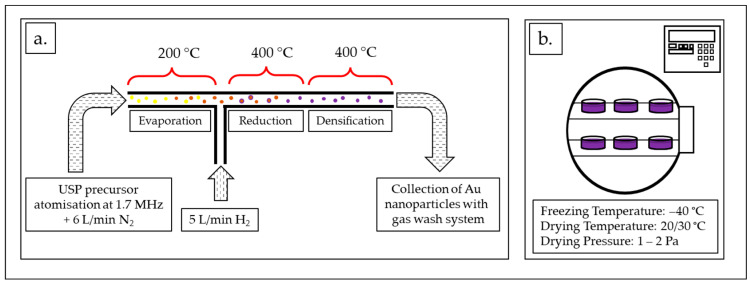
AuNP synthesis process: (**a**) USP synthesis scheme with the process parameters: ultrasound frequency: 1.7 MHz, N_2_ gas flow: 6 L/min, H_2_ gas flow: 5 L/min, temperatures: 200/400/400 °C. (**b**) Freeze-drying of the AuNP suspension obtained using USP with the process parameters: freezing temperature –40 °C, drying temperature: 20/30 °C, drying pressure: 1–2 Pa.

Figure 2a shows an in-house-developed USP device [35]. The AuNPs’ synthesis process using USP was previously described extensively in many scientific articles [24,25,26]. The main elements of the USP device are the ultrasonic generator, the reactor furnace and a system for nanoparticle collection. The sizes of the synthesised AuNPs depend on the ultrasound frequency, which determines the sizes of the aerosol droplets, and the concentration of the dissolved Au in the precursor solution droplets. The frequency used on the ultrasound generator was 1.7 MHz. The USP in-house-developed device has three temperature zones in the reactor. The following temperatures were used in this study: in the first zone, T = 200 °C, while, in the other two zones, T = 400 °C. Nitrogen was used as the carrier gas, with a flow rate of 6 L/min, while hydrogen was used as the reducing gas, with a flow rate of 5 L/min. Hydrogen was required for the decomposition and reduction of the Au–salt ions, which resulted in the formation of AuNPs.

The AuNPs’ collection was performed in glass containers. The collection liquid had an initial volume of 1.0 L. Polyvinylpyrrolidone (PVP) (Sigma-Aldrich, Shanghai, China), with an average molar mass of 40,000 g/mol, was added as the stabilising agent in the collection liquid, with an initial concentration of 4.5 g/L. The stabiliser helps in controlling the size and stability of the AuNPs. During USP synthesis, the volume of the collection liquid increased from the initial 1.0 L to 1.9 L, indicating the deposition and accumulation of AuNPs and water vapour. The total duration of the USP synthesis was 4 h and 30 min.

The produced AuNPs’ suspension was concentrated with rotary evaporation using a Büchi Rotavapor R-300 (Büchi Labortechnik AG, Flawil, Switzerland) at a vapour pressure of 24 mPa, a 200 rpm evaporation flask rotation speed, a bath temperature of 35 °C and an evaporation time 50 min time. The AuNPs suspension was reduced to 600 mL. ICP-OES analysis was used to accurately measure the concentration of Au in the final concentrated AuNPs suspension.

#### 2.2.2. Freeze-Drying of the New AuNPs

The freeze-drying of the concentrated AuNP suspension (600 mL) was carried out using a lyophiliser Labfreez instrument FD-200F SERIES (Labfreez Instruments Group Co., Ltd., Beijing, China) (Figure 2b) over the course of 71 h. The process consisted of 4 h of freezing time at –40 °C and two drying stages, the primary at 20 °C for 12 h and the secondary at 30 °C for 55 h. The resulting dried AuNPs were in the form of a dried PVP cake with a total mass of 5.68 g.

### 2.3. Characterisation Methods

#### 2.3.1. Transmission Electron Microscopy (TEM)

Two types of samples were investigated using TEM: AuNPs from the LFIA conjugate pads and USP-synthesised AuNPs. Sample preparation took place with the drop-casting process, where the shorter pads were deposited on a TEM grid. First, a part of the sample was taken from the conjugate pad and transferred to a centrifuge, which was previously filled with absolute ethanol. Using a dropper, the mixture of ethanol and pads was applied to the TEM grid (film: carbon formvar B, 200 mesh, Cu), and the sample was left on the TEM grid to dry overnight in a desiccator until the start of the analysis. The sample was examined and analysed using imaging, electron diffraction, and elemental composition using a JEM-2100HR (JEOL, Tokyo, Japan) transmission electron microscope (TEM) with an attached energy dispersive X-ray spectrometer (EDS) JED-2300T (JEOL, Japan), at an accelerating voltage of 200 kV, which appeared to cause no damage to the fibre material. EDS analyses were performed at standard conditions of the acquisition method: high resolution, the live time mode, and an acquisition time of 200 s. The probe size was 10 nm or 25 nm at a magnification of ×100,000 [36,37].

#### 2.3.2. Scanning Electron Microscopy (SEM)

The final dried new AuNPs were examined using a scanning electron microscope (SEM), Sirion 400 NC (FEI Sirion 400 NC, FEI Technologies Inc., Hillsboro, OR, USA), with an EDS INCA 350 (Oxford Instruments, Oxford, UK). The samples were prepared by applying the dried AuNPs onto an SEM stub holder with graphite tape.

#### 2.3.3. Ultraviolet-Visible Spectroscopy

Ultraviolet-visible spectroscopy (UV-Vis) was used to evaluate the absorbance of the new AuNPs in comparison to AuNPs prepared with conventional Au salts with USP. The dried AuNPs were resuspended in deionised water (20 mg/mL) and dispersed in an ultrasound bath. A UV-Vis spectrophotometer, Tecan Infinite M200 (Tecan Group Ltd., Männedorf, Switzerland) was used with a special microplate using the following parameters: sample volume: 300 µL, absorbance range: 300–800 nm, wavelength step: 2 nm and five flashes per wavelength.

## 3. Results and Discussion

### 3.1. ICP-OES Results for Au Solutions and AuNP Suspensions

The results of the ICP-OES analysis are given in Table 1. The weight of gold in each solution was calculated based on the obtained ICP-OES results for Au solutions from the conjugate pads after soaking and filtering, as well as for the Au solution after heating. During the precursor preparation, losses of aqua regia were observed and, in turn, Au. During the filtering of the soaked conjugate pads, some of the aqua regia was soaked up by the conjugate pads, which was not retrievable. The results show that 0.99 mg of Au was lost during the heating of the solution. This is attributed to the vigorous boiling of the aqua regia solution at the start of the heating process. Using a higher beaker during the heating process would limit the emission of aqua regia from the beaker. This suggests that using the evaporation of water and decomposition of aqua regia at elevated temperatures should be modest in the recovery process. Heating was used with the aim of reducing the volume and increasing the pH of the resulting Au solution from aqua regia for the subsequent preparation of the precursor in USP (pH > 4) so that there would be no accidental acid damage to the precursor on the membranes of the ultrasonic generator in USP. The starting volume of aqua regia was reduced by 25% during soaking and filtration, and an additional loss of 12.5% was observed during heating. In total, the process used to extract the Au from conjugate pads had a 62.5% recovery rate.

**Figure 3:**
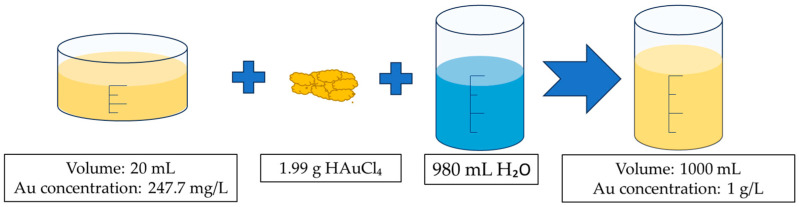
Preparation scheme for the Au precursor. Aqua regia with a leached gold ion solution at a volume of 20 mL, and gold concentration of 247.7 mg/L (determined with ICP-OES) and 1.99 g of HauCL_4_ were added to 980 mL of deionised water and mixed until dissolved to prepare the final precursor solution.

The measured value of Au concentration in the solution after the heating process was too low (247.7 μg/mL) to be used directly as a precursor in the USP synthesis. The process of preparing the final Au precursor solution can be seen in Figure 3.

Research has shown that, due to the loss of Au during the evaporation of the excess water and decomposition of aqua regia, and due to problems with USP at low pH levels, it makes sense to use a smaller amount of aqua regia during the precursor preparation. Smaller quantities of aqua regia would require a progressive rinsing of the conjugate pads since large quantities of them would soak up the aqua regia. A progressive batch process would reduce the loss of aqua regia due to soaking and would, in turn, increase the recovery percentage.

### 3.2. TEM Investigations of AuNPs from Conjugate Pads

**Figure 4:**
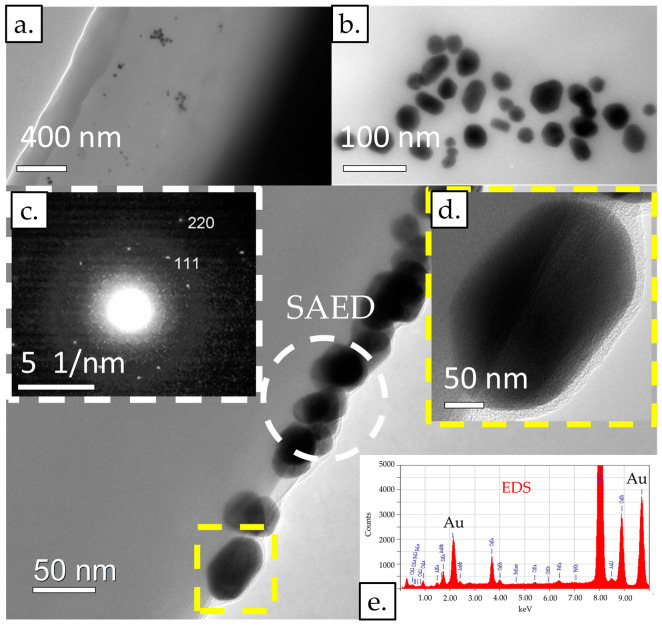
TEM images of an LFIA conjugate pad: (**a**) at lower magnification and (**b**) at higher magnification. (**c**) The electron diffraction (SAED) images of a few AuNPs. (**d**) An image showing an individual AuNP at higher magnification. (**e**) EDS point analysis. Acceleration voltage: 200 kV; bright field BF image; probe size for EDS: 10 nm or 25 nm.

For the TEM and TEM/EDS investigations and analyses, the corresponding areas where the sample was recorded were imaged at multiple sites, each site having multiple images of the same site at different magnifications and comparable identical magnifications at other sites for statistical comparison. Using TEM/EDS analysis, the elemental composition of the sample was determined with spot analyses at several locations for statistical comparison. Some selected results are presented in Figure 4 with the AuNPs marked, observed and detected in the sample.

AuNPs were present in several places on the fibres of the conjugate pad, which were distributed individually in some places, and in some places in agglomerates. The AuNPs were of different morphologies, including rod-shaped, triangular, square-shaped, and most often, polygonal, which indicates their different growth direction. The AuNPs were between 10 nm and 50 nm in size. Using selected area electron diffraction (SAED), the 111 and 220 planes were determined in the area where some AuNPs were present in the agglomerate, which, in addition to elemental analysis using TEM/EDS, confirmed the presence of AuNPs in the sample.

### 3.3. SEM and TEM Investigations of the New Dried AuNPs

The new dried AuNPs were used for the SEM and TEM investigations because they were significantly easier to handle. 

**Figure 5:**
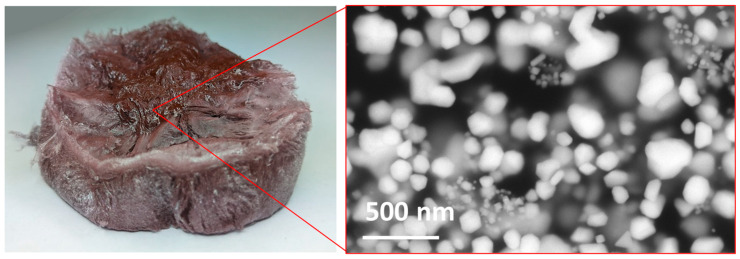
New (brown) dried AuNPs in PVP cakes. A brown area is visible on the surface of the dried AuNP cake, which is usually not seen on dry AuNPs prepared with commercially available metal salts, indicating the presence of particles larger than 100 nm.

The dried AuNPs are shown in Figure 5. A macro examination showed that after drying, there was no moisture in the resulting AuNP/PVP cakes, and the AuNPs were completely dry. The AuNP cakes had good structural stability and a brown colour range; this was not observed in previous AuNP drying experiments, which were synthesised only from metal salts without the addition of recycled material (which was formed by dissolving AuNPs from the conjugated pads). The residue of brown AuNPs was also visible on the sides of the glass containers after removing the cakes.

The SEM and TEM images of the dried AuNPs were analysed to determine their morphology and sizes. The EDS analysis detected elements of gold (83.64 ± 1.87 wt. %) and oxygen (16.36 ± 1.87 wt. %). The oxygen can be attributed to the stabilising agent used, PVP. No other elements were detected as a result of contamination or impurities in the new AuNP samples.

**Figure 6:**
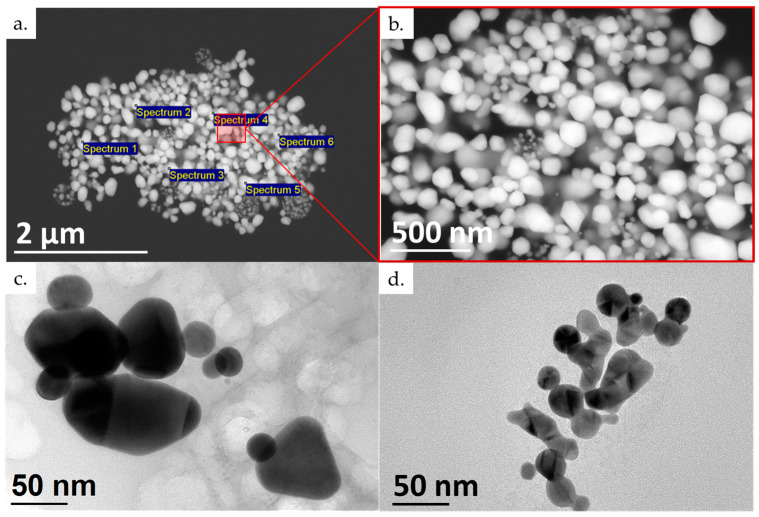
Presentation of the new (brown) dried AuNPs: (**a**) SEM microstructure with EDS spectrum locations; (**b**) detailed SEM microstructure; and (**c**,**d**) TEM of new dried AuNPs. The SEM images were taken with an acceleration voltage of 20 kV, spot size of 3.0, and backscatter electron detector BSE image. The TEM images were taken with an acceleration voltage of 200 kV; bright field BF image.

The morphology investigation showed mostly irregular and spherical shapes with individual particles in the shapes of triangles, pentagons and hexagons, as seen in Figure 6. Their sizes ranged from 10 nm up to 350 nm for the more irregular-shaped particles. From our previous investigations with USP, it is known that very acidic gold solutions for producing AuNPs produce a higher amount of irregularly shaped particles, with sizes of several 100 nm [38]. This is an additional reason for the need to reduce the amount of aqua regia used when dissolving the starting AuNPs from the conjugate pads. These AuNPs also greatly reduce the stability of the produced suspension, promoting a higher rate of particle agglomeration, as seen in the brown spots.

**Figure 7:**
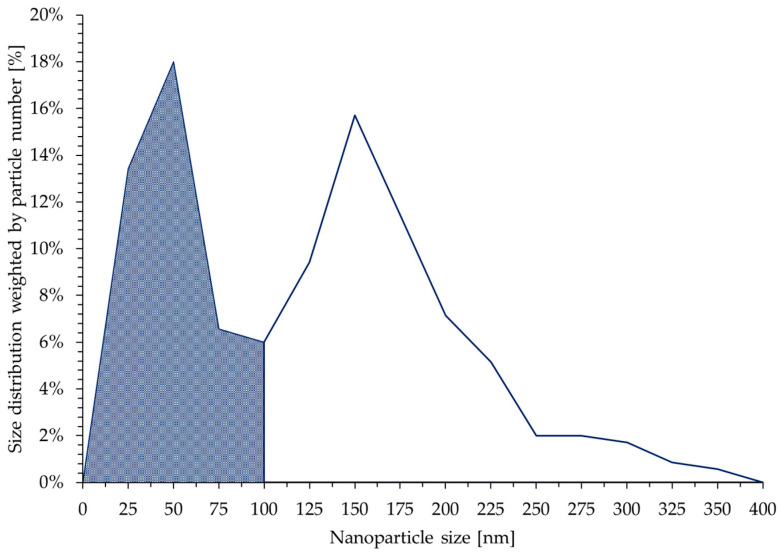
New (brown) dried AuNP size distribution weighted by particle number. In total, 350 AuNPs were analysed. Two distinct peaks can be observed, one at 50 nm and the other at 150 nm. AuNPs with a size smaller than 100 nm represented 44% of the total particles.

The size distribution of the new AuNPs was obtained by measuring the Feret diameter of 350 particles. The distribution is shown in Figure 7. The dark area on the distribution plot represents the fraction of particles whose size is below 100 nm, and this value is 44%. Two distinct peaks can be observed. The peak at 50 nm is narrower compared with the peak at 150 nm. The AuNPs whose size was below 100 nm predominantly consisted of round particles, while the larger particles were irregularly shaped.

The brown areas visible on the dried AuNP cakes are also an indication of an increased rate of agglomeration. The residual brown material on the drying glassware additionally reinforces this argument.

For recycling AuNPs from LFIA using USP and lyophilisation for new dried AuNP synthesis, a route with the use of smaller acid quantities is needed or an approach where the acids are not included in the dissolution of the AuNPs from conjugate pads.

### 3.4. Ultraviolet-Visible Spectroscopy

**Figure 8:**
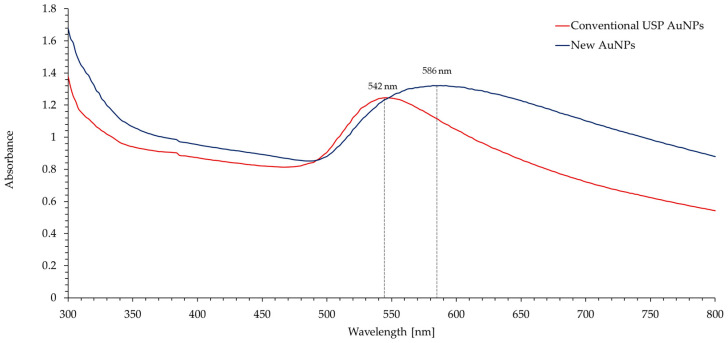
UV-Vis spectra of the new (brown) and conventional AuNPs prepared with USP. The spectra of the new AuNPs compared to the conventional AuNPs show an increased absorbance and indicate a darker brown colour of the nanoparticles.

The obtained UV-Vis spectra, shown in Figure 8, are consistent with the SEM/TEM analyses and the obtained particle size. The absorbance peek in AuNPs is a direct consequence of Mie scattering. Mie scattering is impacted by particle shape and size, the dielectric function of the particles and the refractive index of the surrounding medium. Larger particles increase the absorption of light, and therefore increase the total absorbance. They also shift the absorbance peek towards higher wavelengths [39,40,41]. Both of the aforementioned phenomena can be observed in the UV-Vis spectra of the new (brown) AuNPs and have a direct influence on their brown colour.

## 4. Conclusions

The presence of dispersed AuNPs on the conjugate pads of LFIA was confirmed, and their sizes ranged from 10 nm to 50 nm. 

The recovery of AuNPs from conjugated pads of LFIAs is possible with the use of aqua regia and USP synthesis. Lyophilisation serves as a process to remove water and to obtain dried AuNPs.

Lower amounts of acids should be used for AuNP recovery with USP and lyophilisation since a high acid surplus produces mainly irregular particles with diminished stability. This aspect also detrimentally affects freeze-drying.

The use of USP is beneficial in the context of AuNP recovery from conjugated pads, as the high synthesis temperatures ensure complete degradation of the organic components. In this way, there is no risk of viral infection.

This research provides guidelines for the development of an improved recycling process, not only for AuNPs but also for other metal nanoparticle wastes with reduced or no acid content.

## Figures and Tables

**Table 1:** The concentration of Au in aqua regia solutions prior to and after the heating process and in the AuNP suspension measured using ICP-OES.

Sample	Au (μg/mL)	Volume (mL)	Mass of Gold (mg)
Resulting Au solution from the conjugate pad after soaking and filtering	39.6	150	5.94
Au solution after the heating process	247.7	20	4.95
Final concentrated AuNP suspension after USP	197.5	600	118.5

## Data Availability

Data sharing not applicable.

## Abbreviations

lateral flow immunoassays (LFIAs), gold nanoparticles (AuNPs), optical emission spectrometry with inductively coupled plasma (ICP-OES), ultrasonic spray pyrolysis (USP), polyvinylpyrrolidone (PVP), transmission electron microscopy (TEM), energy dispersive X-ray spectrometer (EDS), scanning electron microscopy (SEM), selected area electron diffraction (SAED), ultraviolet–visible spectroscopy (UV-Vis).
